# Extracellular vesicle derived miRNAs from plasma as promising diagnosis and prognosis biomarkers for neuroblastoma

**DOI:** 10.1016/j.isci.2025.113636

**Published:** 2025-09-25

**Authors:** Duo Zhou, Yilong Wang, Mengying Zhu, Lingjie Li, Jinkai Peng, Yuxiang Hu, Jieni Xiong, Ting Tao, Jinhu Wang, Zhengyan Zhao

**Affiliations:** 1 Department of Genetics and Metabolism, Children’s Hospital, Zhejiang University School of Medicine, Hangzhou, Zhejiang 310052, China; 2 Department of Neurology, Children’s Hospital, Zhejiang University School of Medicine, Hangzhou, Zhejiang 310052, China; 3 Pediatric Cancer Research Center, National Clinical Research Center for Child Health, Hangzhou, Zhejiang, China; 4 Department of Surgical Oncology, Children’s Hospital Zhejiang University School of Medicine, Hangzhou, Zhejiang, China; 5 Cancer Center, Zhejiang University, Hangzhou, Zhejiang, China; 6 National Clinical Research Center for Child Health, Children’s Hospital Zhejiang University School of Medicine, Hangzhou, Zhejiang, China

**Keywords:** Diagnostics, Molecular biology, Oncology

## Abstract

Neuroblastoma (NB) is the most common extracranial solid tumor in children. MYCN amplification remains a critical indicator of high-risk disease and poor prognosis. Small extracellular vesicles (sEVs) carry microRNAs (miRNAs) with promising diagnostic potential. This study identified plasma sEV-derived miRNA biomarkers for NB diagnosis and MYCN status prediction. Using miRNA-seq, we analyzed plasma sEVs from 24 patients with NB (stratified by MYCN status and risk) and 10 healthy controls. Validation was performed in an independent cohort of 87 patients with NB and 47 controls. We identified six miRNAs (miR-150-5p, miR-142-5p, miR-30b-5p, miR-320a-3p, miR-30b, and miR-342-3p) that were significantly dysregulated in patients with NB, all showing excellent diagnostic accuracy (AUC >0.8). Notably, miR-150-5p and miR-342-3p differentiated MYCN-amplified from nonamplified patients (AUC = 0.738). Functional analysis revealed involvement in key NB pathways. Our findings demonstrate that plasma sEV-derived miRNAs represent valuable noninvasive biomarkers for neuroblastoma diagnosis and risk stratification.

## Highlights

• Comprehensive analysis of the sEVs derived miRNAs in the plasma of patients with NB
• Plasma sEV miRNA signature is a potent diagnostic biomarker for NB
• sEV miRNA panel distinguishes MYCN-amplified from MYCN-nonamplified NB
• sEVs-derived miRNAs function as prognostic indicators in neuroblastoma

## Graphical abstract

**Figure:**
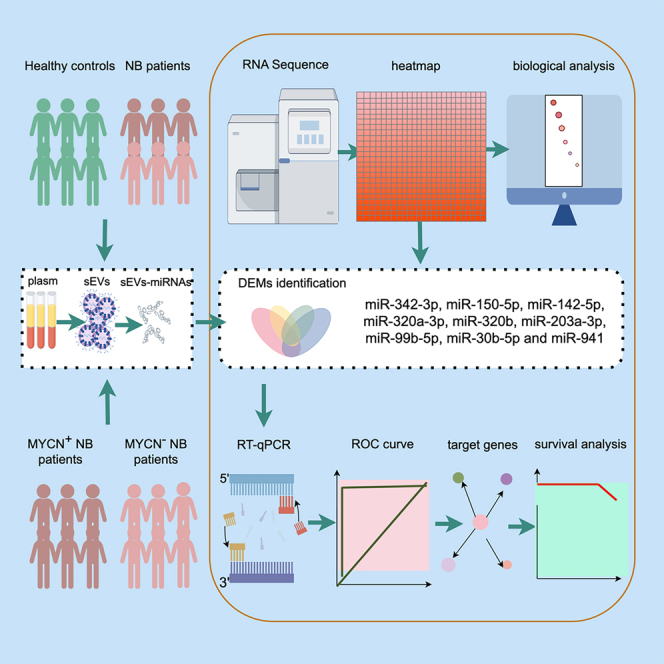

## Introduction

Neuroblastoma (NB), a tumor arising from the developing sympathetic nervous system, is the most common extracranial solid tumor in children, and it accounts for approximately 15% of pediatric cancer deaths.^1^ NB is diagnosed through a combination of laboratory tests, pathologic examinations, and radiographic imaging. According to the International Neuroblastoma Risk Group’s risk classification criteria, NB can be categorized into very-low risk, low-risk (LR), intermediate-risk (IR), and high-risk (HR) groups based on clinical and molecular characteristics prior to clinical treatments.^2^ NB presents a diversity of clinical manifestations and courses depending on the complexity of its biology. Among patients with high-risk neuroblastoma, altered the amplification and expression of the MYCN gene (encoding transcription factor N-MYC) are major hallmarks usually associated with a high risk of recurrence and poor survival.^2^^,^^3^ MYCN regulates the transcription of genes involved in cell motility and extracellular matrix degradation and thereby facilitating tumor cell invasion and metasis.^4^ Besides *its primary function as transcription factor,* MYCN can also act as *a competing endogenous RNA (ceRNA)* for miRNAs to mediate oncogenic features in human malignancies.^5^ Early detection of NB is considered crucial to overcoming this deadly disease of infants and children. The development of new promising, effective diagnosis and treatment biomarkers may contribute to uncover new molecular therapeutic targets and improve patient survival.

Liquid biopsy is a less invasive method with lower costs compared to tissue sampling. During the past ten years, blood-based liquid biopsy applications in oncology have accelerated dramatically with many benefits, including the capability of diagnosing patients with cancer, predicting cancer recurrence, quantifying minimal residual disease, and monitoring treatment responses.^6^ Small extracellular vesicles (sEVs), firstly discovered in the 1980s, are cell-derived membrane extracellular microvesicles with a size ranging from 50 to 150 nm released by cells into the extracellular space and various body fluids including blood.^7^^,^^8^ An increasing number of studies have reported that sEVs can be potentially used as noninvasive diagnostic and prognostic biomarkers for tumors.

MicroRNAs (miRNAs) are non-coding RNA molecules that contain 18-20 nucleotides and act as post-transcriptional regulators of gene expression. The most abundant type of RNA carried in sEVs is miRNA. Compared to freely circulating miRNAs, sEVs provide a stable environment to protect miRNAs from degradation. Numerous studies have demonstrated that expression levels of sEVs-derived miRNAs are deregulated in various types of cancers.^9^^,^^10^^,^^11^ Multiple sEVs-derived miRNAs have shown specific expression patterns in certain cancers, such as miR-363-5p in breast cancer,^12^ miR-18a and miR-221 in hepatocellular carcinoma,^13^ let-7d-3p and miR-30d-5p in cervical cancer, and miR-15a-5p in endometrial carcinoma.^14^ However, there are rare miRNAs identified as diagnosis biomarkers of NB besides miR199a-3p, which is verified with a small cohort.^15^ The role of sEVs-derived miRNAs as noninvasive biomarkers for cancer detection and prognosis has thus become increasingly important.

In the present study, we performed miRNA-Seq using purified plasma sEVs, which showed immunoreactivity against CD63, Tsg101 and HSP 70, but not calnexin, to investigate the sEVs-derived miRNA expression profiles in patients with NB, including patients with MYCN^+^ HR, patients with MYCN^−^ HR, MYCN^−^ IR or patients with LR. We first identified that the expression levels of sEVs-derived miR-150-5p, miR-142-5p, miR-203a-3p, miR-30b-5p, miR-320a-3p, miR-320b, miR-342-3p, miR-941, and miR-99b-5p were significantly different between patients with NB and health controls (HCs). Moreover, we found that the combination of miR-150-5p and miR-142-5p could be used as a potential biomarker to differentiate the MYCN^+^ group from the MYCN^−^ group.

## Results

### Description of the participants’ clinical characteristics

**Figure 1:**
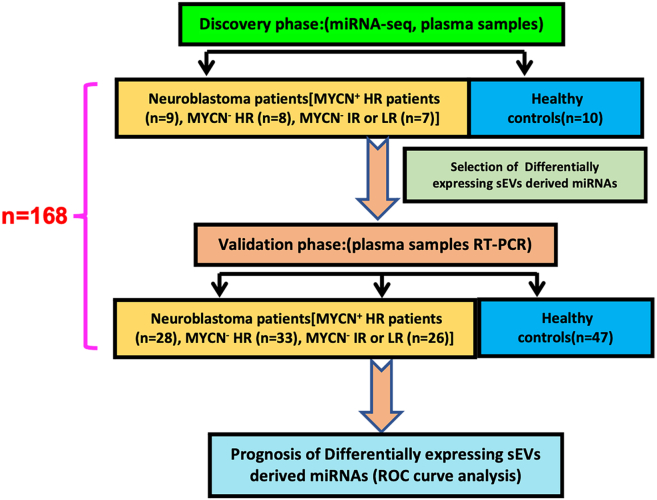
Schematic diagram of the study design 24 NB (9 patients with MYCN^+^ HR, 8 patients with MYCN^−^ HR, and 7 patients with MYCN^−^ IR or LR) patients and 10 healthy children were enrolled to evaluate the expression levels of sEV-derived miRNA by using miRNA-Seq. Additionally, qRT-PCR was used to confirm the levels of differentially expressed plasma sEVs-derived miRNAs in another 87 children in the NB (28 patients with MYCN^+^ HR, 33 patients with MYCN^−^ HR, and 26 patients with MYCN^−^ IR or LR). Finally, the selected sEVs-derived miRNAs were analyzed using a ROC curve analysis to determine whether they can serve as potential markers of NB (n = number of participants). NB, neuroblastoma; HR, high-risk; IR, intermediate-risk; LR, low-risk; sEVs, small extracellular vesicles; miRNA, microRNA; qRT-PCR, quantitative reverse transcription-polymerase chain reaction; ROC, curve receiver operating characteristic curve.

We recruited 168 participants, including 24 children with NB (9 patients with MYCN^+^ HR, 8 patients with MYCN^−^ HR, and 7 patients with MYCN^−^ IR or LR) as well as 10 HCs in the discovery stage, 87 NB children (28 patients with MYCN^+^ HR, 33 patients with MYCN^−^ HR, and 26 patients with MYCN^−^ IR or LR), and 47 HCs during the validation phase in this study (Figure 1). The clinical features of individuals were provided in Table 1. In the screening phase, the mean age of patients with NB and HCs was 3.75 and 5.27 years, respectively, and their mean age was, respectively, 3.94 and 3.21 years, respectively, in the validation phase. The mean age of first diagnosis for patients with MYCN^+^ and patients with MYCN^−^ patients was 3.76 and 3.74 years in the screening phase and was 3.71 and 4.05 years in the validation phase. Our data also demonstrated that 100% of patients with MYCN^+^ and 69% of patients with MYCN^−^ in the validation stage received preoperative chemotherapy. Additionally, HCs enrolled in our research had no history of malignant illnesses, metabolic diseases, neurological illnesses, and inflammatory diseases when collecting blood samples.

**Table 1:** Clinical characteristics features of individuals

	Discovery set			Large-scale validation set		
	NB		HCs	NB		HCs
	MYCN^+^	MYCN^-^		MYCN^+^	MYCN^-^	
NO	9	15	10	28	61	47
Female: Male	4:5	7:8	5:5	9:19	28:33	18:29
Age of first diagnosis ±SD (median)	3.35 ± 1.74(2.79)	3.45 ± 2.08(3.71)	NA	3.17 ± 1.63 (2.59)	3.46 ± 2.67 (3.04)	NA
Age, Mean ± SD (median)	3.75 ± 1.97 (3.48)		5.27 ± 2.67 (4.93)	3.94 ± 2.43 (3.42)		3.21 ± 1.64 (2.96)
	3.76 ± 1.67 (3.09)	3.74 ± 2.19 (3.88)		3.71 ± 1.74 (3.29)	4.05 ± 2.69 (3.32)	
number of patients receiving chemotherapy	9:0(100%)	10:5(67%)	NA	28:0(100%)	42:19(69%)	NA
Survival analysis(Sur:Die:NA)	6:3:0	15:0:0	NA	18:5:5	50:6:5	NA

Age of first diagnosis, Age, used were presented as mean ± standard deviation, rest of data were presented as amount. Abbreviation: NB neuroblastoma, HCs healthy controls, NA not available, Sur:Die:NA survival: die: not available.

### The characterization of plasma-derived small extracellular vesicles from patients with neuroblastoma

**Figure 2:**
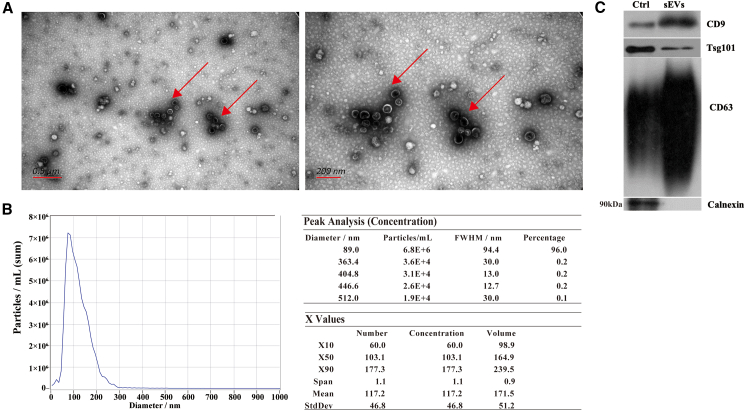
SEVs characterization and quantitative properties (A) A morphological examination of sEVs using a TEM: left, 0.5 μm bar scale; right, 200 nm bar scale. (B) sEVs size distributions and concentrations were measured using NTA. On the right, the particle size distributions and concentrations were depicted. (C) A Western blot analysis was performed to identify positive protein markers (TSG101, CD9 and CD63) and negative protein markers (Calnexin) of sEVs, cell lysates of MSC were used as controls. sEVs, small extracellular vesicles; TEM, transmission electron microscopy; NTA, nanoparticle tracking analysis.

We separated the sEVs-enriched fractions from the plasma of all patients diagnosed with NB and HCs by SEC procedure. Subsequently, we analyzed the morphology and size distribution of these vesicles with TEM and NTA. According to the TEM and NTA results, a majority size distribution of the sEVs measured ranged from 75 nm to 200 nm, which is consistent with sEVs size criteria (Figures 2A and B). By Western blot analysis, CD63, TSG101, and CD9, three of the sEV markers, were detected in the sEV-enriched fractions in plasma samples, while calnexin as a negative marker for sEVs was not detectable (Figure 2C). Our results suggested that circulating sEVs were successfully separated from peripheral plasma samples.

### An analysis of miRNA expression profiles derived from small extracellular vesicles in patients with neuroblastoma and health controls

**Figure 3:**
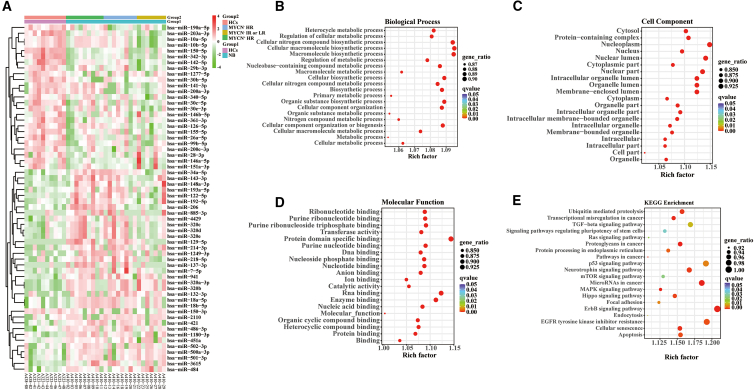
Differential plasma sEVs-derived miRNAs expression analysis of patients with NB (A) Heatmap of DEMs in patients with NB. Right side of the heatmap shows representative miRNAs. Z-scores were displayed on the right using a color bar and colors red and green indicate high and low expression respectively. (B–D) mRNAs targeted by 60 DEMs are represented as bubble plots under GO terms (Biological Process, Cellular Component, Molecular Function). (E) KEGG pathway enrichment bubble plot for 60 DEMs-targeted mRNAs. The q-value is depicted as color code. Bubble size indicates the number of DEMs associated with each pathway. sEVs, small extracellular vesicles; miRNA, microRNA; DEMs, differentially expressed miRNAs; NB, neuroblastoma; HR, high-risk; IR, intermediate-risk; LR, low risk; HCs, healthy controls; mRNAs, messenger RNAs; GO, Gene Ontology; KEGG, Kyoto Encyclopedia of Genes and Genomes.

In order to identify the potential significance of the changes in sEVs-derived miRNAs, sEVs-enriched fractions in 24 plasma samples from patients with NB (9 patients with MYCN^+^ HR, 8 patients with MYCN^−^ HR, and 7 patients with MYCN^−^ IR or LR) and 10 HCs were all sequenced for miRNAs in the first step. (Figure 3A). In comparison with the healthy group, we observed an increase in 34 known miRNAs and a decrease in 26 known miRNAs in the whole patients with NB. We also identified an increase in 35 known miRNAs and a decrease in 19 known miRNAs in the patients with MYCN^+^ HR; an increase in 26 known miRNAs and a decrease in 22 known miRNAs in the patients with MYCN^−^ HR; an increase in 14 known miRNAs and a decrease in 30 known miRNAs in the patients with MYCN^−^ IR or LR in compared with HCs. GO terms in 60 DEMs were then performed to determine their functional significance, 16127 mRNAs targeted by 60 DEMs were enriched in the metabolic process, biosynthetic process, organelle, cell part, intracellular part, protein binding and RNA binding (Figures 3B–3D). KEGG pathway enrichment analysis summarized the top twenty NB-associated signaling pathways, which were also annotated by target mRNAs (Figure 3E). The top twenty significant pathways mainly included the ErbB signaling pathway, microRNA in cancer, Neurotrophin signaling pathway, EGFR tyrosine kinase inhibitor resistance and p53 signaling pathway.

### An analysis of miRNA expression profiles derived from small extracellular vesicles in patients with MYCN^+^ neuroblastoma and patients with MYCN^−^ neuroblastoma

**Figure 4:**
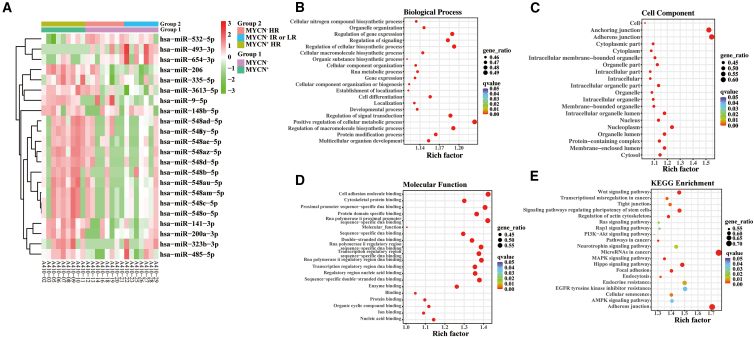
Identification of DEMs between patients with MYCN^+^ and patients with MYCN^−^ (A) Heatmap of DEMs in patients with MYCN^+^ and patients with MYCN^−^. Right side of the heatmap shows representative miRNAs. Z-scores were displayed on the right using a color bar and colors red and green indicate high and low expression, respectively. (B) The bubble plot of Go terms (Biological Process) of mRNAs targeted by the DEMs. (C) The bubble plot of GO terms (Cellular Component) of mRNAs targeted by the DEMs. (D) The bubble plot of GO terms (molecular function) of mRNAs targeted by the DEMs. Bubble size indicates the number of genes associated with each term. E. Bubble plot of KEGG pathway enrichment analyses. The q-value is depicted as a color code. Bubble size indicates the number of DEMs associated with each pathway. DEMs, differentially expressed microRNAs; miRNAs, microRNAs; GO, Gene Ontology; KEGG, Kyoto Encyclopedia of Genes and Genomes.

It has been shown that most malignant neuroblastomas have the amplification of MYCN, which is found in approximately 20% of tumors.^16^ It was shown that the levels of three miRNAs were decreased, whereas those of 19 miRNAs were increased in patients with MYCN^+^ NB in comparison with patients with MYCN^−^NB^-^ (Figure 4A). Patients with MYCN^−^ NB were then divided into two subgroups (subgroup MYCN^−^ HR and MYCN^−^ IR or LR). The trend was similar between subgroup MYCN^−^ HR and MYCN^−^ IR or LR, but the difference was not statistically significant. To evaluate the possible mechanism underlying the identified relations, we subsequently performed GO terms and KEGG pathway enrichment analysis in 22 DEMs. 7810 mRNAs targeted by 22 DEMs were predicted using miRNA databases. GO term analysis provided further insights into characteristics of biological process, cellular component, and molecular function in MYCN^+^ samples compared to MYCN^−^ samples (Figures 4B–4D). In terms of KEGG enrichment analysis results, we presented the top twenty NB-related signaling pathways in Figure 4E, mainly including miRNA in cancer, adherens junction, the Wnt signaling pathway, the signaling pathways regulating pluripotency of stem cells, and the Hippo signaling pathway.

### Small extracellular vesicles-derived microRNAs as diagnostic biomarkers for neuroblastoma in the validation phase

**Figure 5:**
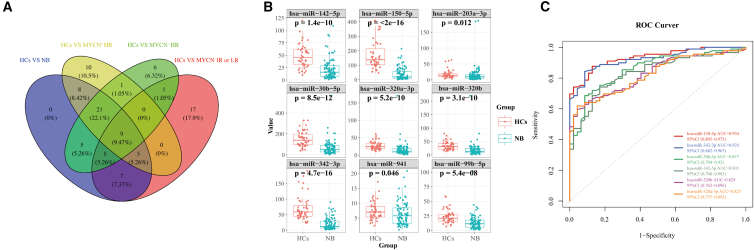
Validation of plasma-sEV-derived miRNAs as potential diagnostic biomarkers using qRT-PCR in 134 independent plasma samples (A) The Venn-diagram for the nine miRNA intersections of four compared groups, namely patients with HC vs. NB, patients with HCs vs. MYCN^+^ HR, patients with HCs vs. MYCN^−^ HR, and HCs vs. MYCN^−^ IR or LR). (B) A comparison of the relative expression levels of nine miRNA intersections in 134 independent validation samples (patients with NB versus HCs). Box dimensions indicate the quartiles for 25–75% accuracy, the middle line represents median values, and the whiskers are minimum and maximum values. (C) Validation of ROC curves for the diagnosis efficiency (patients with NB versus HCs) of each miRNAs individually. The small nucleolar RNA, U6, served as an internal reference. The Student’s t test (two-tailed) was used for the comparative analyses, and the significance threshold was set at 0.05 for each test. sEVs, small extracellular vesicles; miRNA, microRNA; qRT-PCR, quantitative reverse transcription-polymerase chain reaction; NB, neuroblastoma; HCs, healthy controls; ROC curve, receiver operating characteristic curve.

A total of nine common miRNAs (miR-342-3p, miR-150-5p, miR-142-5p, miR-320a-3p, miR-320b, miR-203a-3p, miR-99b-5p, miR-30b-5p and miR-941) were detected among four compared groups in the screening phase (Figure 5A). A total of 87 neuroblastoma children (28 patients with MYCN^+^ HR, 33 patients with MYCN^−^ HR and 26 patients with MYCN^−^ IR or LR) and 47 HCs were enrolled to detect the miRNA levels. We found that the above-mentioned nine miRNAs showed significantly different expression levels (*p* < 0.05) in patients with NB than in HCs (Figure 5B). The six miRNAs with AUC value over 0.8 were presented in Figure 5C. MiR-150-5p showed the diagnostic accuracy with an AUC of 0.934, a sensitivity of 87.6% and specificity of 87.2%; miR-342-3p had the diagnostic accuracy with an AUC of 0.924, a sensitivity of 88.8% and a specificity of 83.0%; miR-30b-5p showed the diagnostic accuracy with an AUC of 0.857, a sensitivity of 85.4% and a specificity of 70.2%; miR-142-5p showed the diagnostic accuracy with an AUC of 0.835, a sensitivity of 84.3% and a specificity of 68.1%; miR-320b exhibited the diagnostic accuracy with an AUC of 0.829, a sensitivity of 65.2% and a specificity of 89.4%; and miR-320a-3p showed the diagnostic accuracy with an AUC of 0.825, a sensitivity of 61.8% and a specificity of 95.7%. Our result suggested miR-150-5p, miR-342-3p, miR-30b-5p, miR-142-5p, miR-320b and miR-320a-3p might have a promising diagnostic accuracy for patients with NB.

### Prognosis efficacy of small extracellular vesicles-derived microRNAs between patients with MYCN^+^ neuroblastoma and patients with MYCN^−^ neuroblastoma in the validation phase

**Figure 6:**
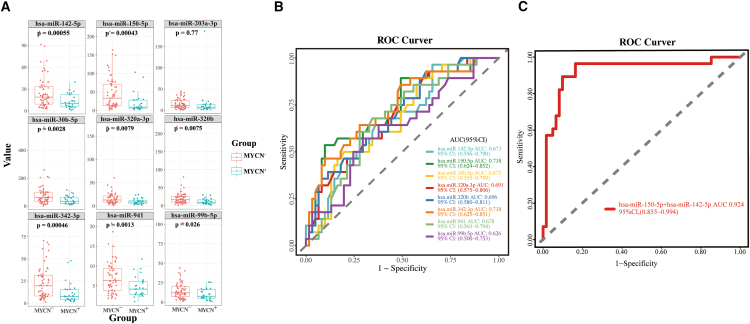
Validation of sEVs-derived miRNAs as potential prognosis biomarkers for discriminating MYCN^+^ and MYCN^−^ groups in plasma samples (A) An analysis of the relative expression levels of nine miRNA intersections in independent validation samples (MYCN^+^ versus MYCN^−^ groups). Box dimensions indicate the quartiles for 25–75% accuracy, the middle line represents median values, and the whiskers are minimum and maximum values. (B) The ROC curve analysis for each of the top six miRNA. (C) The ROC curve for the combined miRNA panel generated using the SVM model. U6 was used as the reference gene for miRNA analysis. T-test was used to determine statistical significance, *p* < 0.05. sEVs, small extracellular vesicles; miRNA, microRNA; ROC curve, receiver operating characteristic curve; SVM model, support vector machine model.

The amplification of MYCN is the most common genetic lesion in patients with sporadic NB and NB with MYCN amplification have limited treatment options and a poor survival rate.^17^ We next investigated the expression changes of nine diagnostic sEVs-derived miRNAs (miR-142-5p, miR-150-5p, miR-203a-3p, miR-30b-5p, miR-320a-3p, miR-320b, miR-342-3p, miR-941, and miR-99b-5p) between patients with MYCN^+^ and MYCN^−^ groups. As showed in Figure 6A, these miRNAs demonstrated significantly different levels (*p* < 0.05) except for miR-203a-3p in comparing patients with MYCN^+^ NB to patients with MYCN^−^ NB. However, single-miRNA ROC curve analysis showed low accuracy with AUC value less than 0.8 (Figure 6B). We performed ROC curve analysis of combined miRNA panel using SVM model. The combined miRNA biomarker panel of miR-150-5p and miR-142-5p achieved an AUC value of 0.924. Our result suggested that two miRNA-panel of miR-150-5p and miR-142-5p could be used to distinguish between patients with MYCN^+^ and patients with MYCN^−^.

### MYCN-related gene interaction network analysis

**Figure 7:**
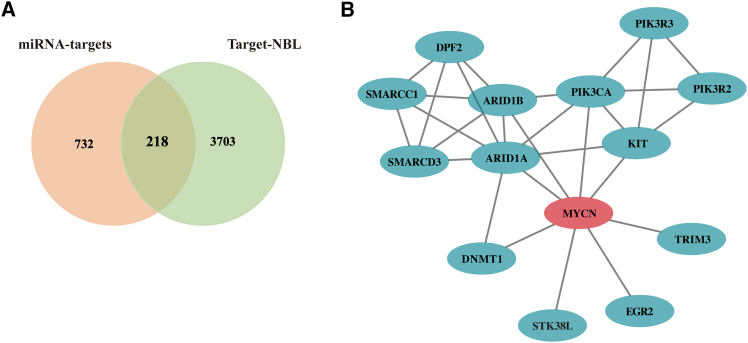
Gene-gene interaction analysis of the MYCN-related genes (A) Intersection of miRNA targets and differential genes in the TARGET database. (B) MYCN^-^related gene interaction network analysis by the STRING database.

Nine miRNA target prediction was performed using miRanda and TargetScan databases. 950 miRNA target genes were obtained. A gene expression dataset TARGET-NB was downloaded from TARGET website. The intersection of these two gene datasets identified 218 genes and 13 MYCN-related genes (Figure 7A) were determined using the STRING tool (Figure 7B). These 13 genes could be used as potential therapeutic targets.

### The validation of correlation between small extracellular vesicles-microRNAs expression and clinical background

To confirm if any correlation exists between sEVs-miRNAs expression and clinical parameters, we analyzed sEVs-miRNAs with age, gender, risk stratifications, clinical stages, and complications. We found no difference in age distribution between the evaluated groups (Figure S1). While we found no significant connection between sEVs-miRNA expression and gender. Similarly, no correlation exits between patients with and without complications (Figure S2). Patients with HR-NB have limited treatment options and a poor survival rate, the overall survival rate is merely 30% after 5 years.^18^ To identify if the candidate miRNAs correlated to risk stratification and clinical stages, we compared the expression levels of these miRNAs in different risk groups and clinical stages. In the validation phase, we directly compared miRNA expression differences between HR and IR/LR groups in the full NB cohort.

As shown in Figure S3, six miRNAs, including miR-142-5p, miR-150-5p, miR-30b-5p, miR-342-3p, miR-941 and miR-99b-5p, had significantly different levels (*p* < 0.05) between HR-NB and IR/LR groups. The expression of these miRNAs was also significantly higher in samples with early stages (L1 or L2 stage) compared to the samples with advanced stage (M stage) (Figure S4).

### Identification of prognostic microRNAs for patients with neuroblastoma

**Figure 8:**
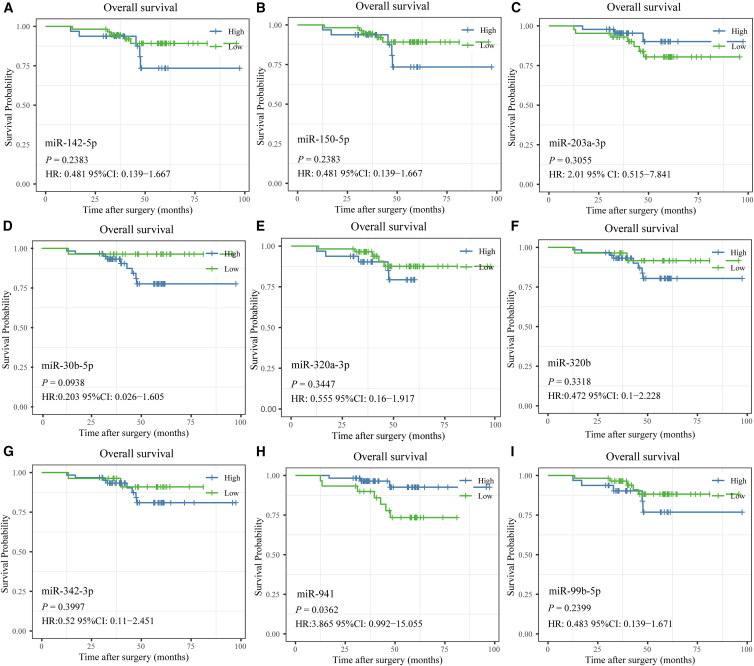
Identification of prognosis-associated miRNAs among nine miRNAs (A–I) K-M survival curve of sEVs-derived miR-150-5p, miR-142-5p, miR-342-3p, miR-203a-3p, miR-320b, miR-320a-3p, miR-30b-5p, and miR-941 in the validation stage.

The expression data from RT-qPCR of nine hallmark miRNAs underwent the Kaplan–Meier (K-M) method. Among them, miR-941 and miR-99b-5p, showed to be associated with overall survival (OS) times (Figure 8). The K-M survival curves indicated that there was a significant difference in prognosis between the low- and high-expression groups of miR-941.

## Discussion

sEVs released by many types of cells, including cancer cells, can be detected in blood, urine, and bronchial fluids, making them potential biomarkers through liquid biopsy.^19^^,^^20^ Plasma-derived sEVs can be sequentially collected over time and analyzed, which could support early diagnosis, prognosis, detection of recurrence, and resistance to therapy of cancers. Recently, numerous studies have demonstrated that sEVs can be used as cancer biomarkers detected in blood samples from patients with glioblastoma, as well as pancreatic, gastric, colorectal, and breast cancers.^21^^,^^22^^,^^23^^,^^24^ A variety of cargoes are carried by sEVs, including DNA fragments, RNA, proteins, and lipids. The miRNAs are the most abundant types of RNA contained in sEVs, due to the fact that miRNAs are protected from RNase-dependent degradation by sEVs. SEVs-derived miRNAs are considered to be more suitable biomarkers.^25^^,^^26^ Recently, a variety of sEVs-derived miRNAs have been associated with the diagnosis and prognosis of cancer, such as sEVs-derived miR-15a-5p from plasma for endometrial cancer, sEVs-derived let-7b-3p and miR-145-3p for colon cancer, sEVs-derived let-7b and miR-18a for myeloma, sEVs-derived miR-21 and miR-1246 for breast cancer, and sEVs-derived miR-1246 and miR-1290 for ovarian cancer.^10^^,^^14^^,^^24^^,^^27^ In previous research, the pathogenesis of NB has been believed to be associated with abnormal miRNA expression profiles, and miRNA expression profiles have been linked to disease severity and prognosis.^28^ Therefore, we hypothesized that miRNAs derived from sEVs in plasma could be useful in NB diagnosis and prognosis. In this study, by using RNA-seq and bioinformatics analysis, we focused on identifying sEV-derived miRNA biomarkers as differential diagnostic biomarkers in NB.

A comprehensive analysis of the sEVs-derived miRNA landscape in patients with NB and HCs was conducted during the discovery phase. Nine miRNAs (miR-342-3p, miR-150-5p, miR-142-5p, miR-320a-3p, miR-320b, miR-203a-3p, miR-99b-5p, miR-30b-5p, and miR-941) detected among four groups in the screening phase were finally selected for further validation in another larger cohort of 134 individuals by qRT-PCR. For miRNA quantification, U6 is extensively used as a reference RNA. Three of these miRNAs, including miR-320a-3p, miR-320b, and miR-941, were shown to be differentially expressed with inconsistent direction (either up- or down-regulation) in two stages. The reasons contributing to the discrepancies observed between discovery and validation set findings are batch effects and the differences in quality control, detection assays, and/or analytical methods between RNA-seq and qRT-PCR. Pieter et al. revealed each of five different RNA-Seq measurement workflows showed a small but specific set of genes with inconsistent measurements between RNA-seq and qRT-PCR, and proposed careful validation of RNA-Seq based expression profiles.^29^

We next identified the AUC values of plasma-sEVs-derived miR-150-5p, miR-142-5p, miR-342-3p, miR-203a-3p, miR-320b-5p, miR-320a-3p, miR-30b-5p, miR-941, and miR-99b-5p ranged from 0.604 to 0.934 with an average of 0.803, and we further obtained four suitable candidates including miR-150-5p, miR-142-5p, miR-342-3p and miR-30b-5p with an AUC of 0.934, 0.835, 0.924 and 0.857, respectively. We also assessed the expression changes of these diagnostic sEVs-derived miRNAs between the MYCN^+^ group and the MYCN^−^ group. Eight miRNAs showed significant difference between the MYCN^+^ and MYCN^−^ groups in terms of expression levels of miRNAs. Although individual miRNAs exhibited low prognostic accuracy, a two-miRNA panel of miR-150-5p and miR-142-5p demonstrates an excellent prognostic performance.

Previous studies have identified dysregulated circulating hsa-miR-150 as a novel potential biomarker for the diagnosis of acute myeloid leukemia,^30^ colorectal cancer,^31^ lymphoma,^32^ and renal cell carcinoma.^33^ The differential expression of circulating miR-142-5p between patients with colon cancer,^34^ renal cell carcinoma,^33^ metastatic melanoma^35^ and their HCs supported its potential role as a diagnostic biomarker. In patients with glioma and glioblastoma , miRNA-342-3p was significantly downregulated compared to normal controls.^36^ Furthermore, sEVs-derived miR-342-3p in blood was demonstrated to have the potential to predict the prognosis of patients with HR-NB undergoing induction chemotherapy.^37^ In addition, miRNA-30b-5p expression levels in plasma were also significantly reduced in patients with breast cancer compared to normal controls.^38^^,^^39^ To our best knowledge, this is the first study to show sEVs-derived miRNAs were significantly dysregulated in patients with NB compared to HCs.

Approximately half of high-risk aggressive tumors are characterized by the amplification of the MYCN gene, an oncogene of the MYC family of transcription factors that controls the expression of many target genes, which in turn regulates fundamental cellular processes, including proliferation, cell growth, apoptosis, and differentiation. MYCN modulates the expression of various miRNAs, and MYCN mRNA expression itself is subject to the control by miRNAs. This homeostasis seems disturbed in neuroblastoma, where MYCN upregulation coincides with the severely increased expression of certain miRNAs. *In vitro* assays of Carlo Dominici et al. provided initial evidence of the contribution of miR-628-3p to MYCN regulation.^40^ The present study evaluated miRNA expression in plasma-derived sEVs from MYCN-amplified as well as non-amplified tumor samples. Our findings provided a set of miRNAs that have the potential to the post-transcriptional regulation of MYCN in neuroblastoma and indicated new targets for MYCN oncogene inhibition. One study has shown that the STK38 modulation of MYCN protein activity and abundance results in the loss of viability of MYCN-amplified neuroblastoma cells.^41^ Moreover, Kit gene has been reported to be preferentially expressed in MYCN-amplified neuroblastoma.^42^ Mutations in the PIK3CA gene were also observed in human neuroblastoma tissues.^43^ A proteomic analysis reveals SMARCC1, ARID1A and ARID1B are mainly related to chromatin remodeling in neuroblastoma.^44^ Another study suggests that EGR2 expression are inversely correlated with MYCN expression in thyroid tissue.^45^ DNMT1 has been proved to be a key regulator in MYCN expression in patients with acute myeloid leukemia.^46^ Further validation research will be conducted in the future.

Despite multimodal therapy options, the prognosis of the patients with HR NB is still poor, with a relatively low 5-year-survival rate.^47^ This study is expected to provide a mechanistic classification of NB through investigating the miRNA expression features in patients from different risk groups, which might improve the clinical management of patients. More intensive therapeutics can be administered in a timely manner to patients with NB in the HR group to evaluate the efficacy of the treatment. Simultaneously, overtreatment might be avoided for patients in IR/LR to reduce side effects associated with treatments. Likewise, clinical stages were also important prognostic factors in patients with NB. In addition to the diagnostic miRNAs, K-M survival curves at the validation stage showed the great efficacy of miR-941 to predict survival rate.

The purpose of the current study was to determine sEV-derived miRNA biomarkers as diagnostic and prognostic candidates for NB. In general, our studies indicated that the sEVs-derived miR-150-5p, miR-142-5p, miR-342-3p, and miR-30b-5p act as potential diagnostic biomarkers to distinguish patients with neuroblastoma from healthy children. In addition, a combined miRNA panel of miR-150-5p and miR-142-5p could be used to differentiate MYCN+ and MYCN- groups. The investigation of survival analysis indicates the great potential of miR-941 to predict the survival rate of patients with NB. As far as we know, this is the largest cohort of patients with NB that were enrolled to evaluate the expression levels of sEVs-derived miRNAs. Importantly, the clinical application of sEVs-derived miRNAs in plasma as diagnostic and prognostic biomarkers of NB would be noninvasive and cost-effective. Independent, prospective, and multicenter studies by blindly validating the potential of these biomarkers are further needed in the future.

### Limitations of the study

We must admit that all of the blood samples from the patients were collected before the surgery, and almost all of the patients in the HR group have a chemotherapy history before the sample collections. Chemotherapy may have an impact on miRNA sequencing results from patient blood samples because it can kill blood and tumor cells, which can affect miRNA expression and release. Additionally, chemotherapy may lead to changes in miRNA splice isoforms and metabolites, further affecting the accuracy and interpretability of miRNA sequencing results. Therefore, our results may be biased since chemotherapy represents a possible impact on miRNA expression. However, the blood sampling was carried out at 3–4 weeks intervals post-chemotherapy completion, and the moment patients had already achieved normal hemogram. We hope it might reduce the impact of chemotherapy on sequencing and validation results. Although some studies have reported several of 13 MYC related genes, knockdown or overexpression of miRNAs and protein expression experiments should be done to validate the value of their potential as therapeutic targets.

## Resource availability

### Lead contact

Further information and requests for resources and reagents should be directed to and will be fulfilled by the lead contact, Yilong Wang (yilongwang@zju.edu.cn).

### Materials availability

All data required to support the claims of this article are included in the main and supplemental information.

### Data and code availability

#### Data

Compliance with national regulations regarding human genetic resources requires that the miRNA sequencing data must be managed under controlled access. Data are hosted at GSA (https://ngdc.cncb.ac.cn/gsa/). Researchers may apply for access by contacting the DAC Chair, Dr. Yilong Wang, at yilongwang@zju.edu.cn. The DAC regulates access in accordance with institutional and national guidelines. Data are to be used solely for research purposes as approved in the data access agreement. Applicants will be required to complete and sign a Data Access Agreement.

#### Code

This study does not report any original code.

#### Additional information

Any additional information required to reanalyze or use the data reported in this article is available from the lead contact upon request.

## Acknowledgments

The authors gratefully acknowledge financial support from the 10.13039/501100001809National Science Foundation of China (grant 32270853), the Special Fund for Incubation of Young Clinical Scientists at Children’s Hospital, Zhejiang University School of Medicine (CHZJU2023YS001), the Program for Young Top-Notch Talents of the same hospital, the National Clinical Research Center for Child Health (Q21co004), and the startup package provided by Children’s Hospital, the Zhejiang University School of Medicine, which together covered running costs, travel grants, and conference attendance.

## Author contributions

Contributor’s statement: D.Z., Y.W., and Z.Z designed the data collection instruments, collected data, performed the initial analyses, and reviewed and revised the article. M.Z., Y.H., J.P., L.L., J.X., F.G., T.T., J.W. coordinated, supervised data collection, and critically reviewed the article for important intellectual content. All authors approved the final article and agreed to be accountable for all aspects of the work. None of the authors has any conflict of interest to disclose. We confirm that we have read the Journal’s position on issues involved in ethical publication and affirm that this report is consistent with those guidelines.

## Declaration of interests

The authors declare no conflicts of interest.

## STAR★Methods

### Key resources table

**Table:** 

REAGENT or RESOURCE	SOURCE	IDENTIFIER
**Antibodies**		

Anti-CD63	Santa Cruz	Cat. #sc-5275; RRID: AB_627877
Anti-CD9	Proteintech	Cat. #60232-1-Ig; RRID: AB_11232215
Anti-TSG101	Abcam	Cat. #ab125011; RRID: AB_10974262
Anti-calnexin	Proteintec	Cat. #10427-2-AP; RRID: AB_2069033

**Critical commercial assays**		

Exosupur column	Echobiotech	Cat#ES9P14e
Amicon® Ultra spin filters	MerckMillipore	Cat#UFC810096
miRNeasy Mini kit	miRNeasy Mini kit	Cat#217004
QIAseq miRNA Library Kit	Qiagen	Cat#331502
PrimeScript RT Reagent Kit	TAKARA	RR037A

**Deposited data**		

RNA-Seq data	This paper	https://ngdc.cncb.ac.cn/gsa-human/s/PUP1VAy0

**Software and algorithms**		

ZetaView PMX 110	Particle Metrix	Zeta View
Bowtie version 1.2.1.1	Bowtie	https://bowtie-bio.sourceforge.net/news.shtml
R version 3.2.3	R language	https://www.r-project.org/
SPSS 28.0.1	SPSS Statistics	https://www.ibm.com/products/spss-statistics
STRING	String database	http://string-db.org/

### Experimental model and study participant details

Researchers at Zhejiang University School of Medicine conducted the study, and the Ethics Committee of the Children’s Hospital, Zhejiang University School of Medicine approved the experiment (2020-IRB-049). All data were acquired only after obtaining written informed consent from the parent or guardian. During the period from January 2018 to April 2021, 111 pediatric patients diagnosed with NB at Children’s Hospital, Zhejiang University School of Medicine based on histopathological findings of tumor biopsies using the International Neuroblastoma Pathology Classification^48^ were included in this study. Patients were staged using clinical, radiographic, and surgical evaluations, in accordance with the International Neuroblastoma Risk Group’s risk classification criteria. No blinding and sample calculation were performed in this study. Inclusion criteria: (i) patients were diagnosed and confirmed histopathologically; (ii) complete imaging and bone marrow aspiration and biopsy findings were required; (iii) patients were clinically evaluated according to International Neuroblastoma Risk Group Staging System (INRGSS); (iv) MYCN amplification was determined by fluorescence *in situ* hybridization (FISH) using standard procedures^49^ in the COG Neuroblastoma Resource Laboratory; (v) treatment according to the guidelines of the Chinese Children’s Cancer Group study NB-2016 (CCCG-NB-2016). Exclusion criteria: the patients with unknown status of the MYCN oncogenes and insufficient diagnostic information were excluded. In addition, 57 age- and gender-matched healthy children were enrolled as a control group in this research. The HCs were from the Examination Center of Children’s Hospital, Zhejiang University School of Medicine with no malignant illnesses, no infectious diseases, metabolic diseases and no neurological illness. Hemolyzed samples were not included in this study. The sample size evaluation was performed using post hoc power analysis.^50^^,^^51^ The analysis results suggested the sample size of RNA-Seq for a power of 0.8 and effect size of 1.1 to achieve a statistical significance level of 0.05 in HCs vs. NB. And effect size of sample size in MYCN+ vs. MYCN- is 1.24, accordingly. The expected effect sizes for calculating the required sample size of transcription-polymerase chain reaction (qRT-PCR) were 0.51 in HCs vs. NB and 0.65 in MYCN+ vs. MYCN- based on a power of 0.80 for a significance level of 0.05. Power was determined with GPower 3.1 (www.ats.ucla.edu/stat/gpower/). To quantify the strength of the differences, Cohen’s d was calculated as a measure of effect size: 0.2 considered a small effect size, 0.5 a medium effect size, and 0.8 a large effect size.

For the discovery phase of this study, RNA-Seq was used to determine sEVs-derived miRNA levels from plasma samples of NB patients (9 MYCN^+^ HR patients, 8 MYCN^−^ HR patients and 7 MYCN^−^ IR or LR patients) and 10 HCs. Further, qRT-PCR was used to verify the levels of differentially expressed sEVs-derived miRNAs in plasma samples from a validation cohort of additional 87 NB patients (28 MYCN^+^ HR patients, 33 MYCN^−^ HR patients and 26 MYCN^−^ IR or LR patients) and 47 HCs.

### Method details

#### Blood collection and isolation of sEVs

Peripheral blood samples from NB patients and HCs were collected in EDTA tubes. The peripheral blood was centrifuged at 3000 g for 10 min at 4°C to separate the plasma and stored it at −80°C for the sEVs isolation. Size exclusion chromatography (SEC) with ultrafiltration has the advantages of effective recovery rate as well as a high specificity and has been reported to have excellent performance in isolation of sEVs.^52^^,^^53^^,^^54^ Hence, sEVs were isolated from the plasma using SEC method with minor change.^55^ 1 mL of plasma was first filtered with a 0.8-μm membrane and then the filtered sample was covered on an Exosupur column (Echobiotech, Beijing, China; Cat. #ES9P14e). The samples were then eluted with further 0.01 M PBS and a total of 2 mL eluate fractions were collected from SEC column according to the manufacturer’s instructions.Then, sEV fractions were obtained using the ultracentrifugation method (4°C, 150000 g, 2 h). Fractions were concentrated at 4000 g for 1 min by 100 kDa molecular weight cut-off Amicon Ultra spin filters (*MerckMillipore*, Darmstadt, Germany; Cat. #UFC810096).

#### Nanoparticle tracking analysis (NTA)

Vesicles from the suspension liquid with concentrations ranging from 1 × 10^7^/mL to 1 × 10^9^/mL were estimated using ZetaView PMX 110 (Particle Metrix, Meerbusch, Germany) armed with a 405 nm laser source to confirm the size and quantity of the separated vesicles. With an NTA software ZetaView (version 8.02.28; Particle Metrix, Meerbusch, Germany), a 60-s video was captured at a rate of 30 frames per second and the movements of the vehicles were recorded.

#### Transmission electron microscopy (TEM)

Plasma sEVs samples of 10 μL were incubated on a copper mesh at 20°C for 1 min, treated for 1 min with uranyl acetate solution, and then washed with sterile distilled water. After removing the water, the samples were kept for 2 min under incandescent light, and images were captured on a H-7650 transmission electron microscope (Hitachi Ltd., Tokyo, Japan).

#### Western blot assay

Western blot analysis was performed to assess the presence of sEVs markers (10% SDS-polyacrylamide gel electrophoresis; 50 μg of protein per lane). The primary antibodies CD63 (Santa Cruz, CA, USA; Cat. #sc-5275), CD9 (Proteintech, Rosemont, IL, USA; Cat. #60232-I-Ig), and TSG101 (Abcam, Cambridge, MA, USA; Cat. #125011) were used as EV markers, while a negative control was performed with calnexin (Proteintech, Rosemont, IL, USA; Cat. #10427-2-AP) protein marker for sEVs recognition. The control group received cell lysates extracted from mesenchymal stem cell (MSC). Briefly, the protein concentration of sEVs was measured by BCA Protein assay kit. According to the quantitative results, the quantity of sEVs in each sample was calculated, and an appropriate amount of 5X sodium dodecyl sulfate (SDS) buffer was added, vortexed and mixed. Then the protein electrophoresis was performed after denaturation in 95°C water for 5 min. After electrophoresis was completed, the separation gel was removed, and the target protein in the gel was transferred to a polyvinylidene difluoride (PVDF) membrane. After blocking with a 3% bovine serum albumin (BSA) blocking solution, the membranes were incubated with the primary and secondary antibodies, developed, fixed, and exposed.

#### Library preparation and sequencing

The miRNeasy Mini kit (miRNeasy Mini kit, Frederick, MD, USA; Cat. #217004) was used to extract and purify RNA samples from sEVs in accordance with the manufacturer’s instructions. The RNA Nano 6000 Assay Kit of Agilent Bioanalyzer 2100 System (Agilent Technologies, CA, USA) was used to evaluate the concentration and purity of RNA samples. 3 ng of RNA from each sample was used as the input material for small RNA libraries preparation. Sequencing libraries were created using a QIAseq miRNA Library Kit (Qiagen, Frederick, MD, USA; Cat. #331502), as directed by the manual, and index codes were added to attribute sequences to each sample. During cDNA synthesis and PCR amplification, a unique molecular index (UMI) of reverse transcription (RT) primers were designed to analyze the quantification of miRNA expression levels. Then the Agilent 2100 BioAnalyze technology and qPCR were applied to evaluate the quality of the library. The clustering analysis for the index-coded samples was performed using acBot Cluster Generation System and TruSeq PE Cluster Kitv3-cBot-HS (Illumina, San Diego, CA, USA), as recommended by the manufacturer. After cluster production, the library preparations were sequenced on an Illumina NovaSeq 6000 platform and paired-end reads were generated at EchoBiotech Co. Ltd., Beijing, China.

#### The identification of differentially expressed miRNAs

Initially, miRNA sequencing data were processed using in-house Perl scripts. Raw data was cleaned up by removing adapter reads, ploy-N reads, and low-quality reads. Raw data was trimmed and cleaned after removing sequences shorter than 15 nucleotides or longer than 35 nucleotides. Additionally, the calculation was conducted in Q20, Q30, GC-content and sequence duplication level of the clean data. The downstream analysis was based on clean and high-quality data. The Bowtie software was used to align Clean Reads with Silva database, GtRNAdb database, Rfam database and Repbase database, respectively. Then the repeats and ncRNAs, such as ribosomal RNA (rRNA), transfer RNA (tRNA), small nuclear RNA (snRNA), and small nucleolar RNA (snoRNA) were filtered. The remaining reads were used to detect known miRNA and predict new miRNA by comparing with known miRNAs from miRbase and human genome (GRCh38). Expression matrix of quantified UMI counts of miRNAs was normalized to transcript per million (TPM) and calculated to relative log expression via the EdgeR package. For each sample, almost 21.61 M raw reads were generated using an illumina NovaSeq 6000 platform. In total, 1479 known miRNAs were detected by the sequencing and miRNAs with a median TPM of less than ten were excluded from further analysis to avoid bias caused by relatively low expression levels. FC > 1.5 or ≤0.67 and *p* < 0.05 were the screening conditions for DEMs analysis. Heatmap, volcano diagram, and Venn was generated to visualize DEMs using R package pheatmap, ggplot2, and Venn Diagram, respectively (R version 3.2.3).

#### Bioinformatic analysis

The miRNA target genes were predicted using TargetScan and miRanda databases. To analysis the potential functions of significantly dysregulated miRNAs, the R package clusterProfiler(v4.0)was used to perform functional annotation and enrichment analysis of Gene ontology (GO) and Kyoto Encyclopedia of Genes and Genomes (KEGG) database for the miRNA target genes. For the three major categories in the GO database (biological processes, cell components, and molecular functions), as well as the KEGG pathways, the 20 significant, pivotal terms/pathways are displayed. The gene expression for TARGET-NB were downloaded from the TARGET database. The intersection genes between miRNA predicted targets and TARGET-NB were uploaded to STRING (http://string-db.org/) and MYCN-related gene interactions were analyzed.

#### qRT-PCR analysis of miRNA expression

The miRNeasy Mini kit (Qiagen, Frederick, MD, USA; Cat. #217004) was selected to isolate and refine the total sample of sEVs-derived miRNAs in plasma. Reverse transcription of total RNA products into complementary DNA (cDNA) was performed using PrimeScript RT Reagent Kit (Perfect Real Time) (TAKARA, RR037A). Target genes expression levels were detected by TaqMan probe through qPCR. 2 μL of cDNA as a template was subjected to each PCR reaction. All primer and probe sequences are shown in Table S1. Relative expression values are normalized to RNU6-1 (U6) snRNA (internal control) and calculated using the 2^−ΔΔCt^ method. All experiments in validation phase were conducted in triplicates and then the mean value was calculated.

### Quantification and statistical analysis

SPSS 28.0.1 software was used to compare clinical data of NB patients to HCs with Student’s t-tests. Plasma-sEVs-derived miRNA expression levels were determined using the 2^−ΔΔCt^ method, and *p*-value significance analysis was performed using two-tailed student’s t-tests, and the results were reported with R package “ggplot2”. We did not exclude any outliers in our data analysis. A receiver operator characteristic (ROC) curve analysis of single miRNA was conducted with the pROC package. The values of area under the curve (AUC) were then calculated for evaluating the diagnostic efficacy of selected DEMs. We performed ROC analysis of combined miRNA panel based on support vector machine (SVM) classifier in R language. Normality was tested in R with the function shapiro.test. *p* ≥ 0.05 indicates that the data are normally distributed. The vast majority of miRNA expression data from RNA-Seq is abnormally distributed. The miRNA expression data from RT-qPCR are all normally distributed. No test for outliers was conducted on the data. Kaplan–Meier survival analysis was applied to the prognosis survival analysis. The optimal cutoff value of the expression of miRNAs was determined by “surv_cutpoint” in the R package “survminer”.

### Additional resources

This study is not a clinical trial.
