# Supplementary material for: Extracellular vesicle derived miRNAs from plasma as promising diagnosis and prognosis biomarkers for neuroblastoma
Source: iScience. 2025 Sep 25;28(11):113636. doi: 10.1016/j.isci.2025.113636 (PMC12549392; doi:10.1016/j.isci.2025.113636)
Supplement: Document S1. Figures S1–S4, Table S1, Data S1, and S2 [file mmc1.pdf]

**iScience, Volume *28***

## Supplemental information

# Extracellular vesicle derived miRNAs from plasma as promising diagnosis and prognosis biomarkers for neuroblastoma

**Duo Zhou, Yilong Wang, Mengying Zhu, Lingjie Li, Jinkai Peng, Yuxiang Hu, Jieni Xiong, Ting Tao, Jinhu Wang, and Zhengyan Zhao**

# Supplemental Materials

## Figures

### Figure S1

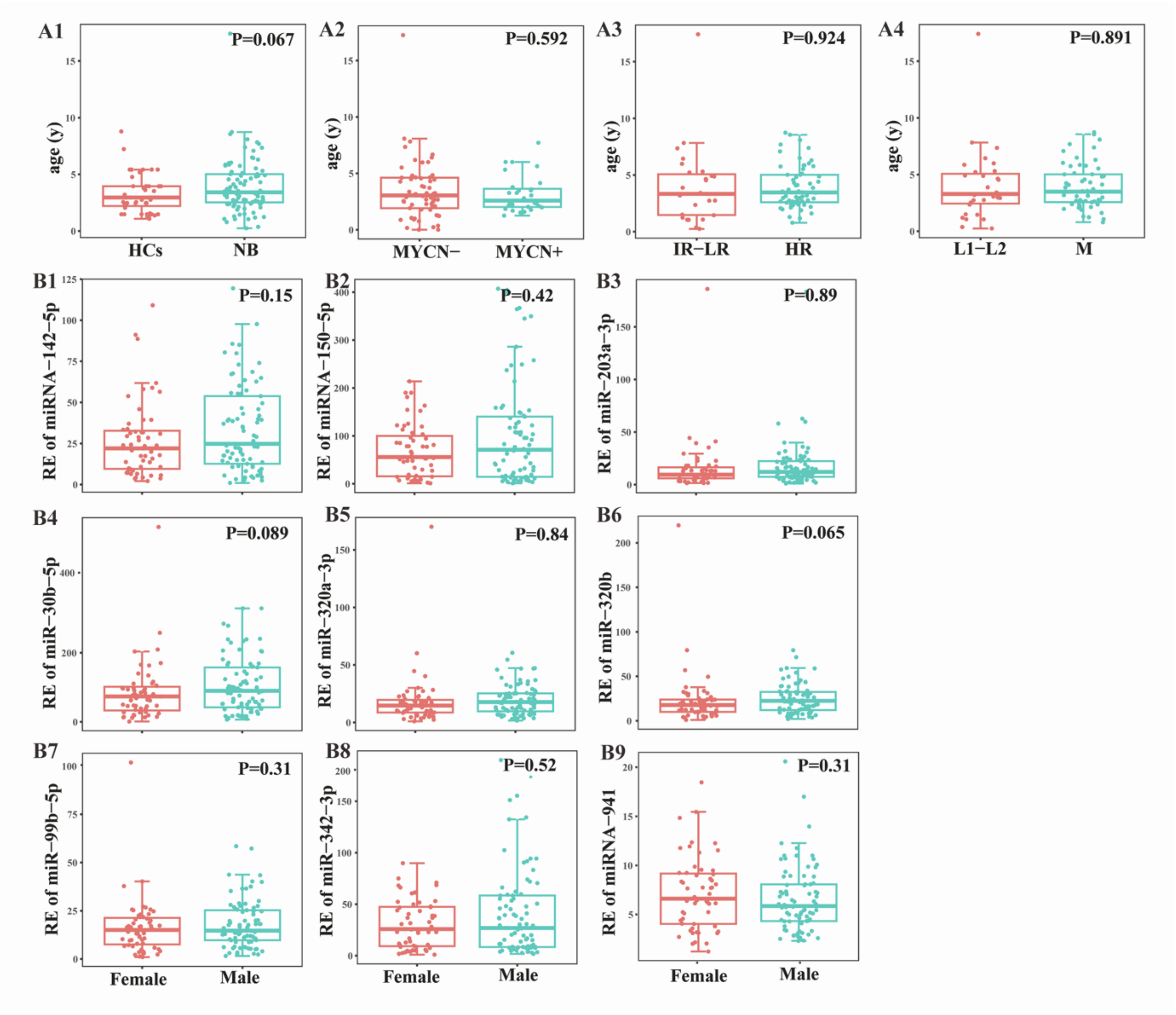


**Figure S1. Effects of age and sex on expression of sEVs-derived miRNAs.**

A1-4. The age differences across the patients in different risk groups and clinical stages, and with or without MYCN amplification. B. Expression of candidate miRNAs in female patients compared to male patients. Box dimensions indicate the quartiles for 25-75 % accuracy, the middle line represents median values, and the whiskers are minimum and maximum values. The corresponding *P*-values are listed in the upper right corner of each panel.

**Fig S2**

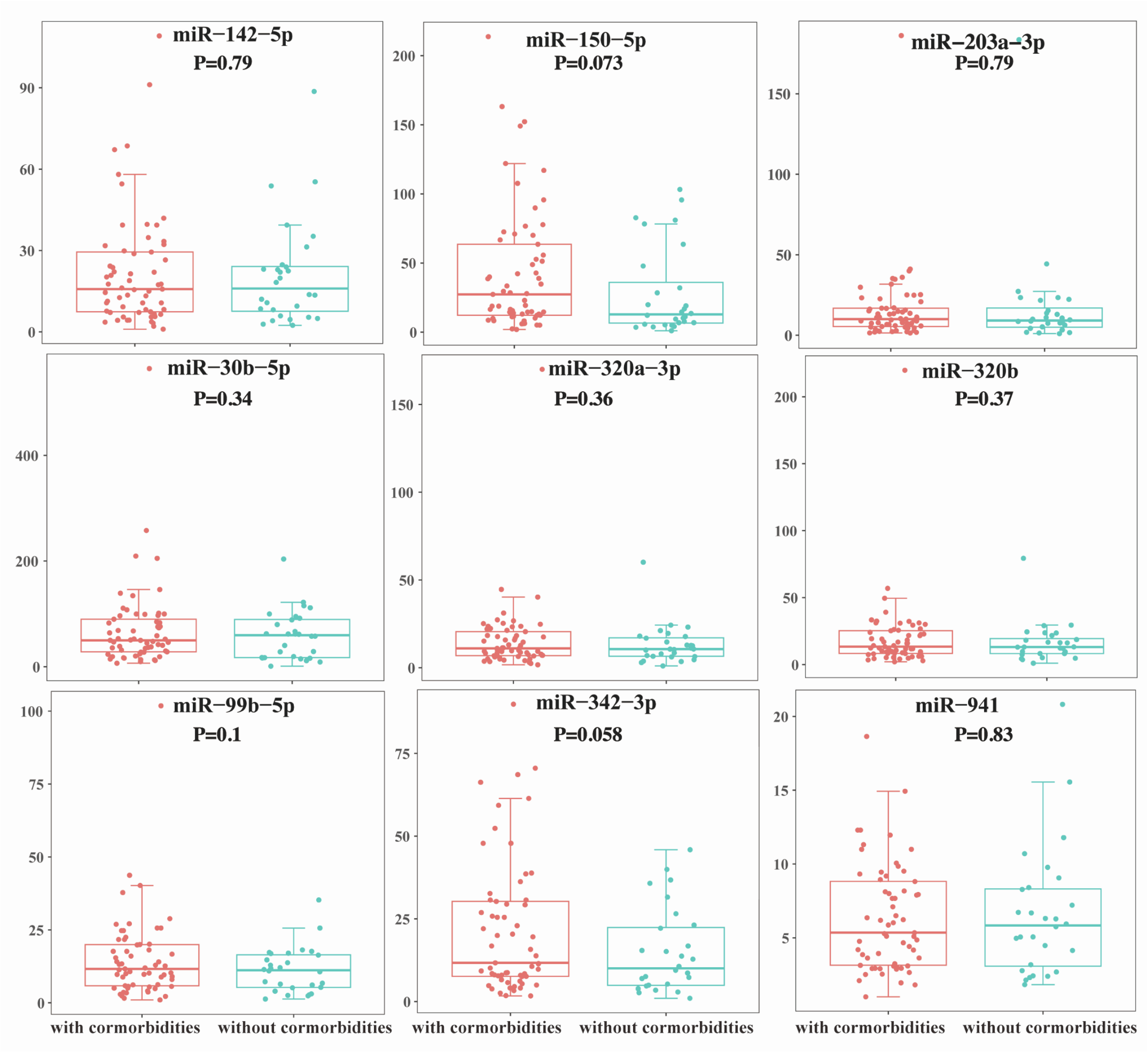


**Figure S2. The comorbidity effects on expression of nine miRNAs.**

Box dimensions indicate the quartiles for 25-75 % accuracy, the middle line represents median values, and the whiskers are minimum and maximum values. The corresponding *P*-values are indicated on top of each panel.

**Figure S3**

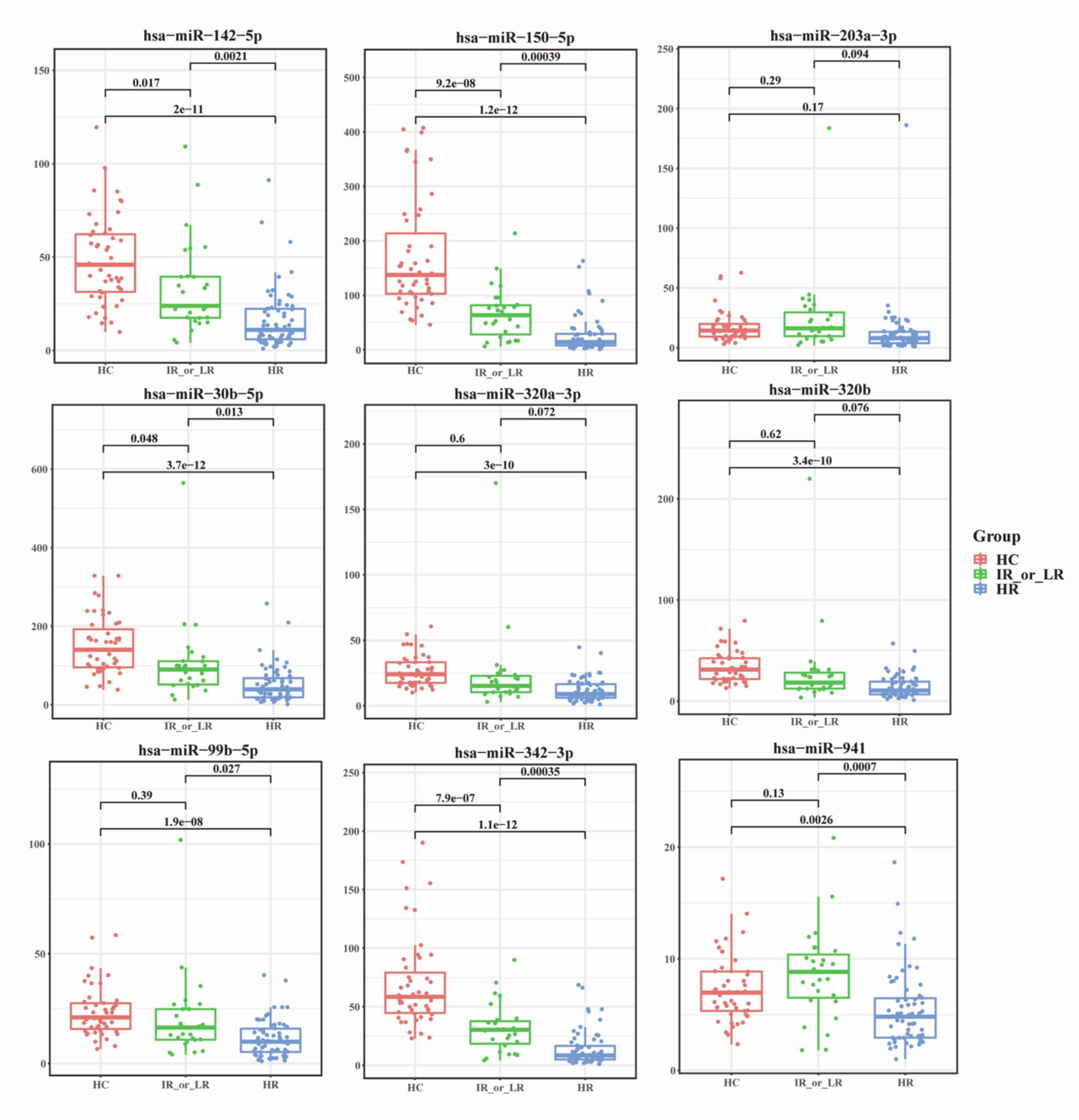


**Figure S3. Validation of sEVs-derived miRNAs as potential biomarkers for discriminating HR and IR/LR groups in plasma samples.**

An analysis of the relative expression levels of sEVs-derived miR-150-5p, miR-142-5p, miR-342-3p, miR-203a-3p, miR-320b, miR-320a-3p, miR-30b-5p and miR-941 in independent validation samples (HR versus IR/LR groups).

**Figure S4**

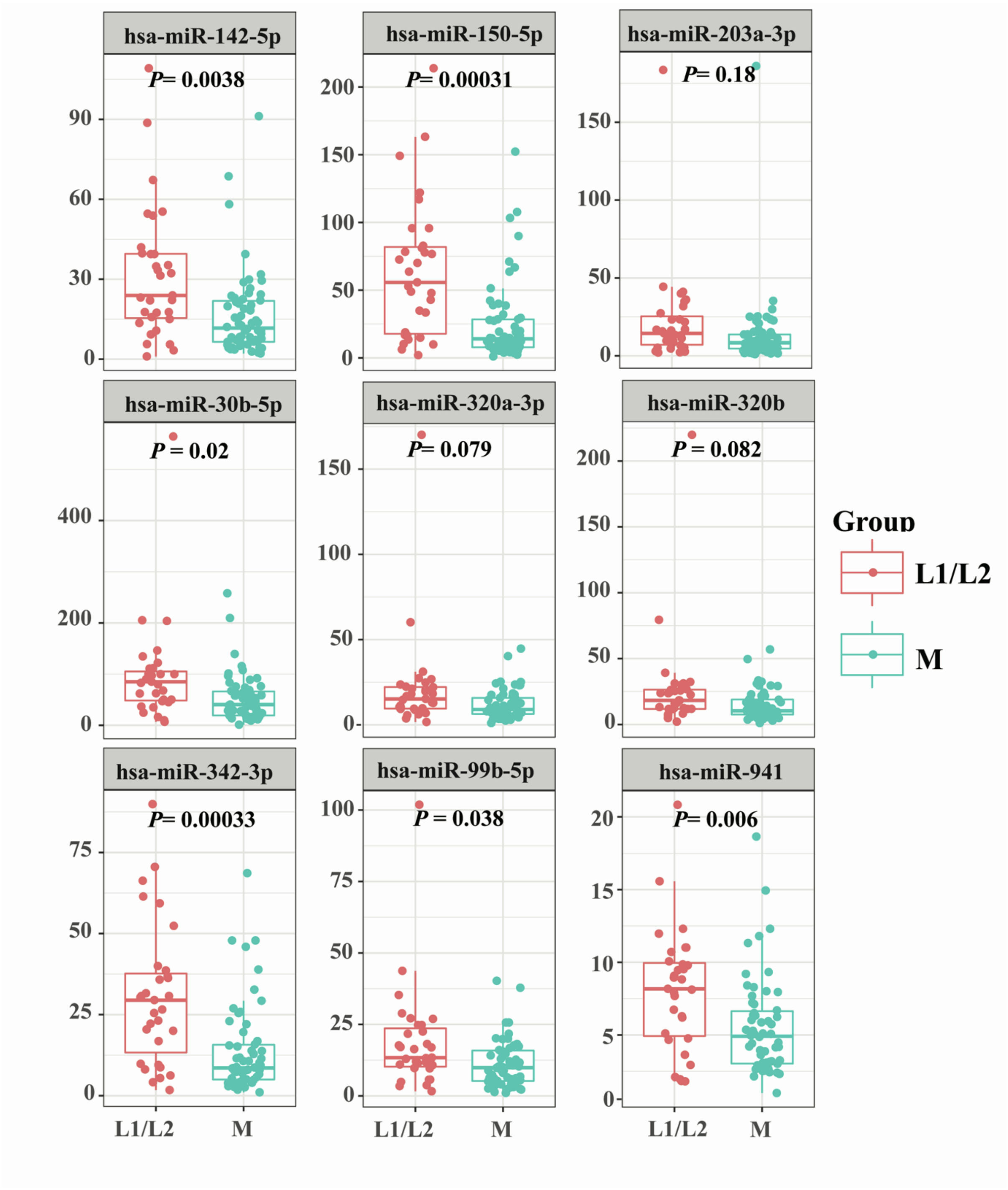


**Figure S4. Validation of sEVs-derived miRNAs as potential biomarkers for discriminating M and L1/L2 groups in plasma samples.**

**Tables**

**Table S1. Sequences of the oligonucleotides used for PCR**

| Primer/probe | Sequence (5′-3′) |
|---|---|
| U6-RT | AACGCTTCACGAATTTGCGT |
| U6-S | CTCGCTTCGGCAGCACA |
| U6-A | AACGCTTCACGAATTTGCGT |
| U6 probe | FAM-AGAAGATTAGCATGGCCCCTGCGCA-BHQ1 |
| hsa-miR-142-5p-RT | GTCGTATCCAGTGCAGGGTCCGAGGTATTCGCACTGGATACGACAGTAGT |
| hsa-miR-142-5p-F | CGCCGCATAAAGTAGAAAGC |
| hsa-miR-142-5p-P | FAM-TTCGCACTGGATACGACAGTAGTG-BHQ1 |
| hsa-miR-150-5p-RT | GTCGTATCCAGTGCAGGGTCCGAGGTATTCGCACTGGATACGACCACTGG |
| hsa-miR-150-5p-F | CGGTCTCCCAACCCTTGTA |
| hsa-miR-150-5p-P | FAM-TTCGCACTGGATACGACCACTGG-BHQ1 |
| hsa-miR-203a-3p-RT | GTCGTATCCAGTGCAGGGTCCGAGGTATTCGCACTGGATACGACCTAGTG |
| hsa-miR-203a-3p-F | ACGGCGTGAAATGTTTAGGAC |
| hsa-miR-203a-3p-P | FAM-TTCGCACTGGATACGACCTAGTG-BHQ1 |
| hsa-miR-320a-3p-RT | GTCGTATCCAGTGCAGGGTCCGAGGTATTCGCACTGGATACGACTCGCCC |
| hsa-miR-320a-3p-F | ACGCAAAAGCTGGGTTGAGA |
| hsa-miR-320a-3p-p | FAM- TTCGCACTGGATACGACTCGCCC -BHQ1 |
| hsa-miR-320b-RT | GTCGTATCCAGTGCAGGGTCCGAGGTATTCGCACTGGATACGACTTGCCC |
| hsa-miR-320b-F | ACGCAAAAGCTGGGTTGAGA |

| | |
|---|---|
| hsa-miR-320b-P | FAM-TTCGCACTGGATACGACTTGCCC-BHQ1 |
| hsa-miR-342-3p-RT | GTCGTATCCAGTGCAGGGTCCGAGGTATTCGCACTGGATACGACACGGGT |
| hsa-miR-342-3p-F | AGCTCTCACACAGAAATCGC |
| hsa-miR-342-3p-P | FAM-CGCACTGGATACGACACGGGT-BHQ1 |
| hsa-miR-99b-5p-RT | GTCGTATCCAGTGCAGGGTCCGAGGTATTCGCACTGGATACGACCGCAAG |
| hsa-miR-99b-5p-F | AGCACCCGTAGAACCGA |
| hsa-miR-99b-5p-P | FAM-TTCGCACTGGATACGACCGCAAG-BHQ1 |
| hsa-miR-421-RT | GTCGTATCCAGTGCAGGGTCCGAGGTATTCGCACTGGATACGACGCGCCC |
| hsa-miR-421-F | AGCGGCATCAACAGACATTAAT |
| hsa-miR-421-P | FAM-TTCGCACTGGATACGACGCGC-BHQ1 |
| hsa-miR-30b-5p -RT | GTCGTATCCAGTGCAGGGTCCGAGGTATTCGCACTGGATACGACAGCTGA |
| hsa-miR-30b-5p-F | TACCAGGTGTAAACATCCTACAC |
| hsa-miR-30b-5p-P | FAM-TTCGCACTGGATACGACAGCTGA-BHQ1 |
| hsa-miR-941-RT | GTCGTATCCAGTGCAGGGTCCGAGGTATTCGCACTGGATACGACGCACAT |
| hsa-miR-941-F | CCCGGCTGTGTGCAC |
| hsa-miR-941-P | FAM-TTCGCACTGGATACGACGCACAT-BHQ1 |

# Data S1: Data access agreement

## GSA-Human 数据访问协议

## DATA ACCESS AGREEMENT （GSA-Human）

（国家基因组科学数据中心, National Genomics Data Center）

本数据访问协议是国家基因组科学数据中心为用户（即数据请求者）和数据递交者（即生产者）提供的数据“申请-审核”协议，系在使用者和递交者相互认可的基础之上签署的约束性条款。数据使用者和递交者均同意受本条款和条件的约束。

The DATA ACCESS AGREEMENT OF GSA-HUMAN (mention as the AGREEMENT below) is the “request-approval” agreement made by the National Genomics Data Center, which is to bound the data producers and users on the basis of mutual recognition. The data producers and users both agree to be bounded by the terms and conditions in the agreement.

## 一、定义（DEFINITIONS）

**数据**：本协议中的数据特指GSA-Human系统中存储的人类遗传资源组学数据信息。

DATA: The data in this agreement specifically refer to the human genetic omics data in the GSA-Human datasets.

**数据递交者**：亦称“数据生产者”，指向GSA-Human系统中递交数据的人员或研究团队。

DATA SUBMITTER: Also call data producer, refer to the individual or research team of the data submitting to GSA-Human.

**用户**：亦称为“数据请求者”，指通过GSA-Human系统注册，申请获取数

据的研究人员。

DATA USER：Also call data requester，refer to registered individual researcher requesting access to GSA-Human datasets.

**研究参与者**：用户所在实验室或研究单元内的参与研究人员或团队。

RESEARCH PARTICIPANT: refer to the researcher or team in the labs or research units of the DATA USER.

**项目**：用户申请数据将要开展的研究项目。

PROJECT: refer to the research program to which the data will be applied.

**研究目的**：指用户将要利用申请获得的数据拟开展的研究目标，包括但不限于开展疾病机理、治疗、表型、人群队列、理论与模型、工具与方法等研究。

RESEARCH PURPOSE: Shall mean research that is seeking to advance the understanding of genetics and genomics, including, but not limited to researches on mechanism of disease, treatment, phenotype, cohort, theory, model, methods and tools.

**出版物**：包括但不限于在印刷刊物、电子期刊、评论、书籍、海报和其他书面和口头研究报告中发表的文章。

PUBLICATIONS: Includes, but not limited to articles published in print journals, electronic journals, reviews, books, posters and other written and verbal presentations of research.

**所获数据**：特指用户通过GSA-Human系统获得的组学数据。

OBTAINED DATASETS: refer to the OMICS data downloaded from GSA-Human.

**数据管理委员会（DAC）**：指数据递交者指定的数据管理与审核工作组，负责承担用户申请数据使用权限的审批。

DAC：refer to the data access committee appointed by the data submitters and responsible for the approval of the rights of the data using.

## 二、权利与义务（**Rights and Obligations**）

1. 用户同意遵守本次申请所涉及数据集（附录I）的相关使用规定，承认数据生产者的贡献，并在使用所获数据产生的所有报告或出版物中适当体现。

Data user agrees to obey the terms and conditions while using these obtained datasets under the application (described in Appendix I) for the research purpose in the project. This includes but is not limited to recognizing the contribution of the data Producers and including a proper acknowledgement in all reports or publications resulting from the use of these obtained datasets.

2. 用户同意遵守《中华人民共和国人类遗传资源管理条例》中的相关规定和条款。

Data user agrees to follow the Regulations on the management of the People's Republic of China on the Administration of Human Genetics.

3. 用户同意遵守《国家基因组科学数据中心人类遗传资源数据共享政策》的相关规定和条款。

Data user agrees to follow the terms and conditions of the Principle for the Access of Human Genetic Resource Data in NGDC made by National Genomics Data Center.

4. 用户同意始终对所获数据保密，并采用适当的措施防止数据泄露。特别地，用户承诺不使用所获数据来损害相关研究人员的成果。

Data user agrees to keep the data confidential, and use proper measure to prevent data disclosure. In particular, data user promises not to use obtained

datasets to jeopardies the results of relevant researchers.

5. 用户同意仅将所获数据用于附录 II 所示的研究目的，如若用于其他研究目的，则需重新申请数据使用权。

Data user agrees only to use the accessed data for the research purpose (described in Appendix II). Data User should submit another separate application for the other research purpose even using the same accessed data.

6. 用户可以将所获数据分享给本项目的研究参与者，但禁止将所获数据向任何未授权的第三方传递与扩散。

Data user agrees only to transfer or disclose these obtained datasets, in whole or part, or any material derived from these obtained datasets, to the authorized personnel such as the participants of the declared project in the data application. Data user agrees not to transfer and spread the data to the unauthorized third parties.

7. 用户同意将所获数据与相关信息及时向所在单位报备，接受所在单位的监督和管理。

Data user agrees to report to the user institution for the data accessing and be willing to receive the supervision of the user institution meanwhile.

8. 用户所获数据的知识产权属于数据生产者，用户同意不就这些数据提出知识产权主张，也不能以任何理由和方式申请这些数据的知识产权。

The intellectual property of obtained datasets belongs to data producer. Data user agrees not to make intellectual property claims on these obtained datasets, nor to apply for intellectual property in any ways or reasons.

9、 用户同意一旦所获数据不再用于附录 II 的研究，应将其销毁/丢弃，除非有义务按照审计或法律要求保留数据以供存档。

Data user agrees to destroy/discard the data, once it is no longer used for the project listed in APPENDIX II, unless obliged to retain the data for archival purposes in conformity with audit or legal requirements.

10、如发现用户未按相关约定使用所获数据，数据生产者可以书面通知用户终止本协议。如果本协议因任何原因终止，用户所在机构将被要求销毁所持有的任何数据，包括副本和备份副本。

Data producer may terminate this agreement by written notice to data user should data user is found to use the obtained datasets without following relevant rules. If this agreement is terminated for any reason, data user will be required to destroy any data held, including copies and backup copies.

11. 用户同意数据生产者可能需要不时修改本协议的条款。如果需要更改，数据生产者将通过 GSA-Human 数据平台实施，用户可以选择接受更改或终止协议。

Data user agrees the regulations concerning the multiple modifications of the data on the GSA-Human platform by the data producer and user has the right to accept the changes or terminate the agreement.

12. 本协议（以及因本协议或其形成而产生的任何性质的争议、争论、诉讼或索赔）应根据中国法律进行解释和管辖。

This Agreement (and any dispute, controversy, lawsuit or claim of any nature arising out of this agreement or its formation) shall be interpreted and governed by the laws of China.

13. 用户同意在使用所获数据从事研究后获得的再生数据，应优先提交至GSA-Human平台进行数据发布与共享。

Data user agrees to submit the newly regenerated data of the research to GSA human platform for data release and sharing.

14、用户同意在使用所获数据的过程中，发生的任何问题、风险等均与GSA-Human平台及平台开发部门和单位无关。

Data user agrees that there is no relationship between the data platform, data developing departments and its institutions of GSA-Human and the occurred risks or problems when user accessing the data on GSA-Human.

15、用户和数据递交者如若使用本协议的电子签名模式，则电子签名后的协议同样具有法律及约束效力。

Data user agrees that the electronic signature mode of this agreement has the same legal and binding effect.

16. 国家基因组科学数据中心对本协议的具体条款具有解释权利。

The final interpretation right to the specific terms of this agreement lies on the National Genomics Data Center.

## 三、确认与签字( Agreement Confirmation with signature )

用户（数据申请者）所在机构( Data User's Institution )

| | |
|---|---|
| 机构签名/章( Signature ) | |
| 机构名称( Institution name ) | Children's Hospital, Zhejiang University School of Medicine |
| 国家/地区( Country/District ) | China |
| 联系人( Contactor ) | 汪一[illegible] |
| 联系人邮件( Email ) | Yilong wang @ zju.edu.cn |
| 日期( Date ) | 2025. 3. 25 |

数据申请信息及用户（申请者）签字：

**Data User's information and signature:**

我确认我已阅读并理解本协议，并遵守本协议的相关约定。

I confirmed that I am fully aware that agreement and promise to follow its regulations and conditions.

| | |
|---|---|
| 用户签字( signature ) | 汪一[illegible] |
| 研究题目( Project name ) | Single - cell landscape revealed immune characteristics associated with infection disease |
| 研究期限( Research period ) | 2027-03-21 |
| 邮件地址( email ) | yilongwang@zju.edu.cn |
| 日　　期( Date ) | 2025. 3. 25 |

数据管理委员会（DAC）：

我确认我已阅读并理解本协议，并代表数据管理委员会（DAC）同意该用户使用数据。

I have read the agreement and am fully aware its regulations and conditions and representative of DAC to approve the data using application of the user.

| | |
|---|---|
| DAC签名( DAC signature ) | 卜军 |
| 邮件地址( email ) | yufei8828@gmail.com |
| 日　　期( Date ) | 2025-04-14 |

-----------------------以下内容为GSA-Human系统自动生成-----------------------

# 四、 附录（APPENDIX）

## 附录I：数据集详细信息（根据递交至GSA-Human系统的数据自动生成）

## APPENDIX I – DATASET DETAILS (automatically create after requestor complete the information on GSA-Human system)

（1）数据集所属的研究（Study）名称

The research (study) name of the dataset

HRA009014,Integrating scRNA and T-cell/B-cell Receptor Sequencing with Mass Cytometry Reveals Dynamic Trajectories of Human Peripheral Immune Cells from Birth to Old Age

（2）数据集详细信息（Study详细信息）

Datasets details (Research or Study details)

Comprehensive understanding of the evolution of the immune landscape in humans across the entire lifespan at single-cell transcriptional and protein levels, during development, maturation, and senescence is currently lacking. We recruited a total of 220 healthy volunteers from Shanghai Pudong Cohort (NCT05206643), spanning 13 age groups from 0 to over 90 years, and profiled their peripheral immune cells through single-cell RNA-sequencing coupled with single T-cell/B-cell receptor sequencing, high-throughput mass cytometry, bulk RNA-sequencing, and flow cytometry validation experiments.

（3）研究领域的具体限制

Specific limitations on areas of research or study

General purpose use;Non-profitable use;

## 附录II：项目详情（根据用户录入GSA-Human系统中请求信息自动生成）

## APPENDIX II – PROJECT DETAILS (automatically create after the requestor complete the information on GSA-Human systerm)

（1）项目名称
project name

HRA009014

（2）项目目标（不超过200字）
Brief abstract of the Project in which the Data will be used (less than 200 words)

获得正常组的单细胞数据补充对照组

（3）采取的方法与技术（不超过200字）
Description on the methods and technologies of the project (less than 200 words)

获得正常组的单细胞数据作为对照，进行单细胞数据分析

（4）所需数据集的详细信息（GSA-Human项目与数据集检索号）
Details of dataset requested i.e., GSA-Human Study and Dataset Accession Number

Study ID: HRA009014

Title: Integrating scRNA and T-cell/B-cell Receptor Sequencing with Mass Cytometry Reveals Dynamic Trajectories of Human Peripheral Immune Cells from Birth to Old Age

Data S2: Clinical and demographic details

| No. | sex | age | race/ancestry/ethnicity |
|---|---|---|---|
| Screening phase | | | |
| 1 | F | 6 66 | East Asian |
| 2 | F | 6 00 | East Asian |
| 3 | F | 5 32 | East Asian |
| 4 | M | 5 06 | East Asian |
| 5 | M | 4 91 | East Asian |
| 6 | M | 4 62 | East Asian |
| 7 | F | 4 45 | East Asian |
| 8 | M | 4 16 | East Asian |
| 9 | M | 4 00 | East Asian |
| 10 | M | 2 98 | East Asian |
| 11 | F | 2 79 | East Asian |
| 12 | M | 2 68 | East Asian |
| 13 | M | 2 04 | East Asian |
| 14 | F | 1 77 | East Asian |
| 15 | F | 1 70 | East Asian |
| 16 | M | 1 59 | East Asian |
| 17 | F | 1 05 | East Asian |
| 18 | M | 7 16 | East Asian |
| 19 | M | 4 55 | East Asian |
| 20 | M | 3 71 | East Asian |
| 21 | F | 2 49 | East Asian |
| 22 | F | 1 00 | East Asian |
| 23 | F | 0 65 | East Asian |
| 24 | M | 0 61 | East Asian |
| Validation phase | | | |
| 1 | F | 2 04 | East Asian |
| 2 | F | 1 77 | East Asian |
| 3 | F | 2 23 | East Asian |
| 4 | F | 2 79 | East Asian |
| 5 | F | 3 47 | East Asian |
| 6 | F | 1 85 | East Asian |
| 7 | F | 3 27 | East Asian |
| 8 | F | 6 01 | East Asian |
| 9 | F | 7 71 | East Asian |
| 10 | M | 1 53 | East Asian |
| 11 | M | 1 26 | East Asian |
| 12 | M | 1 70 | East Asian |
| 13 | M | 1 59 | East Asian |
| 14 | M | 2 10 | East Asian |
| 15 | M | 2 15 | East Asian |
| 16 | M | 2 30 | East Asian |
| 17 | M | 2 38 | East Asian |
| 18 | M | 2 32 | East Asian |
| 19 | M | 1 98 | East Asian |
| 20 | M | 3 58 | East Asian |
| 21 | M | 3 27 | East Asian |
| 22 | M | 3 77 | East Asian |

| | | | |
|---|---|---|---|
| 23 | M | 4 14 | East Asian |
| 24 | M | 3 04 | East Asian |
| 25 | M | 3 50 | East Asian |
| 26 | M | 5 32 | East Asian |
| 27 | M | 6 00 | East Asian |
| 28 | M | 5 64 | East Asian |
| 29 | F | 1 92 | East Asian |
| 30 | F | 2 68 | East Asian |
| 31 | F | 2 23 | East Asian |
| 32 | F | 1 78 | East Asian |
| 33 | F | 2 99 | East Asian |
| 34 | F | 3 04 | East Asian |
| 35 | F | 3 16 | East Asian |
| 36 | F | 2 10 | East Asian |
| 37 | F | 2 89 | East Asian |
| 38 | F | 6 66 | East Asian |
| 39 | F | 6 19 | East Asian |
| 40 | F | 0 91 | East Asian |
| 41 | M | 1 05 | East Asian |
| 42 | M | 1 80 | East Asian |
| 43 | M | 2 09 | East Asian |
| 44 | M | 2 85 | East Asian |
| 45 | M | 3 81 | East Asian |
| 46 | M | 3 24 | East Asian |
| 47 | M | 2 68 | East Asian |
| 48 | M | 2 50 | East Asian |
| 49 | M | 2 55 | East Asian |
| 50 | M | 4 84 | East Asian |
| 51 | M | 4 30 | East Asian |
| 52 | M | 4 64 | East Asian |
| 53 | M | 4 45 | East Asian |
| 54 | M | 5 52 | East Asian |
| 55 | M | 4 27 | East Asian |
| 56 | M | 6 00 | East Asian |
| 57 | M | 4 64 | East Asian |
| 58 | F | 2 67 | East Asian |
| 59 | F | 4 80 | East Asian |
| 60 | F | 4 62 | East Asian |
| 61 | M | 0 36 | East Asian |
| 62 | F | 8 07 | East Asian |
| 63 | F | 0 23 | East Asian |
| 64 | F | 2 28 | East Asian |
| 65 | F | 2 30 | East Asian |
| 66 | F | 1 29 | East Asian |
| 67 | F | 3 37 | East Asian |
| 68 | F | 4 55 | East Asian |
| 69 | F | 4 49 | East Asian |
| 70 | F | 3 87 | East Asian |
| 71 | F | 6 40 | East Asian |

| | | | |
|---|---|---|---|
| 72 | M | 0  01 | East Asian |
| 73 | M | 1  22 | East Asian |
| 74 | M | 0  88 | East Asian |
| 75 | M | 1  27 | East Asian |
| 76 | M | 1  00 | East Asian |
| 77 | M | 2  49 | East Asian |
| 78 | M | 3  07 | East Asian |
| 79 | M | 3  18 | East Asian |
| 80 | M | 3  25 | East Asian |
| 81 | M | 0  76 | East Asian |
| 82 | M | 3  71 | East Asian |
| 83 | M | 4  72 | East Asian |
| 84 | M | 0  16 | East Asian |
| 85 | M | 5  97 | East Asian |
| 86 | M | 7  34 | East Asian |
| 87 | F | 7  82 | East Asian |
| 88 | F | 17  25 | East Asian |
| 89 | F | 0  01 | East Asian |
